# Inhomogeneous Microstructure Evolution of 6061 Aluminum Alloyat High Rotating Speed Submerged Friction Stir Processing

**DOI:** 10.3390/ma16020579

**Published:** 2023-01-06

**Authors:** Yuchen Peng, Zonghua Xie, Changchao Su, Yuefang Zhong, Zushan Tao, Dongyang Zhuang, Jiahui Zeng, Hongqun Tang, Zhengbing Xu

**Affiliations:** 1 School of Resources, Environment and Materials, Guangxi University, Nanning 530004, China; 2 Key Laboratory of High Performance Structural Materials and Thermo-Surface Processing, Education Department of Guangxi Zhuang Autonomous Region, Guangxi University, Nanning 530004, China; 3 State Key Laboratory of Featured Metal Materials and Life-Cycle Safety for Composite Structures, Nanning 530004, China

**Keywords:** 6061 aluminum alloy, high rotating speed submerged friction stir processing, strength-ductility synergy, plastic–elastic interface, Inhomogeneous microstructure

## Abstract

An inhomogeneous microstructure induced by high rotating speed submerged friction stir processing (HRS-SFSP) on 6061 aluminum alloy was researched in detail.The microstructures of the aluminum alloy processing zone were characterized by electron backscattered diffraction (EBSD) and transmission electron microscope (TEM) qualitatively and quantitatively.The results show that the recrystallization proportion in the inhomogeneous structure of the processing zone is 14.3%, 37.8% and 35.9%, respectively. Different degrees of grain deformation can affect the dislocation and lead to the formation of a plastic–elastic interface. At the same time, the second-phase particles in the processing zone were inhomogeneity and relatively, which further promotes the plastic–elastic interface effect. The plastic–elastic interface can significantly improve the strength of aluminum alloy, whileat the same time, rely on recrystallized grains to provide enough plasticity. When the rotation speed was 3600 r/min, the strength and ductility of the aluminum alloy after HRS-SFSP were increased by 48.7% and 10.2% respectively compared with that of BM. In all, the plastic–elastic interface can be formed by using high rotating speed submerged friction stir processing, and the strength-ductility synergy of aluminum alloy can be realized at the plastic–elastic interface.

## 1. Introduction

Traditional processing will lead to local inhomogeneity of mechanical properties at the processing interface, easily produce defects and lead to performance degradation [1]. At present, the traditional processing methods mainly include liquid phase method and solid-state processing.Compared with the liquid-phase method, the solid-state processing can effectively avoid the local clusters at the liquid–solid interface in the solidification process and achieve the effect of homogenization of the structure [2]. Severe plastic deformation (SPD) technology is a feasible solid-state processing technology, which can obtain excellent strength by refining grain [3,4]. However, SPD technology often has inhomogeneous deformation [5,6]. This inhomogeneity makes the material form an inhomogeneous structure at the micro level. Under the action of external force, the structure will be unstable, and then deformation or even crack will occur. For FCC metals (such as aluminum), multiple slip systems will start during plastic deformation [7]. Therefore, different mechanical properties will be obtained under different strain conditions.

Friction stir processing (FSP) was improved by R.S. Mishra et al. [8] and is based on friction stir welding (FSW). Its principle is the same as that of FSW. It consists of a shaft shoulder tool with a pin tool, which rotates and moves forward at the same time. The friction between the shaft shoulder and the sheet is used to soften the metal in the processing zone, and the microstructure of the plate changes significantly under the shear action of the pin tool, and then changes the technology of various properties of materials [9,10,11,12]. There is no doubt that FSW or FSP, as an advanced solid-state connection and processing technology, has a good application prospect. However, the traditional FSP has the problem of uneven local mechanical properties. SIMAR et al. [13] found that the strain hardening ability of HAZ and SZ is different, and the location of tensile fracture always occurs in HAZ. In order to solve the problem of inhomogeneous mechanical properties in the processing zone caused by traditional FSP, we proposed a new method:while the pin tool was rotating at a high rotating speed (3600 r/min), we conducted the whole experiment under water. Through the rapid cooling of water, the heat input of processing was reduced and the growth of the processing zone was prevented [14]. We define this process as high rotating speed submerged friction stir processing (HRS-SFSP). The strength-ductility synergy was realized by forming a plastic–elastic interface to store defects [15].

Strength and ductility have always been mutually exclusive properties in metallic materials. It is a complex problem to overcome the strength ductility synergism, which depends on the particle size, crystal orientation, interface matching degree and inhomogeneity of the matrix and the second phase [16,17,18,19,20]. For a long time, researchers have been committed to designing processing methods and regulating various microstructures to improve the strong plastic synergy in metals. HRS-SFSP has the characteristics of low heat input and large plastic deformation. Due to the excellent processing properties of 6xxx aluminum alloys, they have been widely used in various large plastic deformation and 6xxx aluminum alloy is a good representative as the research material [21,22,23]. The microstructure information of 6061 aluminum alloy after HRS-SFSP was analyzed qualitatively and quantitatively by EBSD and TEM. The forming mechanism of strength-ductility in HRS-SFSP aluminum alloy was revealed, which provided guidance for improving the mechanical properties of the processing interface.

## 2. Materials and Experimental Setup

### 2.1. Material Preparation

**Figure 1:**
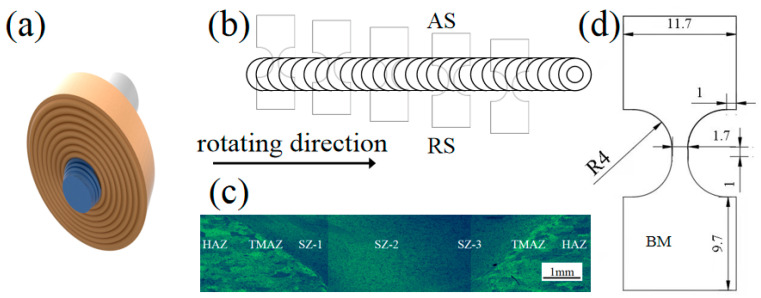
(**a**) Shape of pin tool; (**b**) Sampling position of tensile sample; (**c**) Section optical morphology of HRS-SFSP and (**d**) Size of tensile sample.

The material used in the study is 6061-O aluminum alloy sheet. The main ingredients are shown in Table 1, melting point is 617 °C. A 2 mm pin tool with a top diameter of 3.5 mm and a tip diameter of 2.5 mm with a shoulder diameter of 10 mm was used for the HRS-SFSP, as shown in Figure 1a. Three different rotating speeds were used: 1800 r/min, 3600 r/min and 5400 r/min, the travelling speed was fixed at 60 mm/min and the depth of pressing was 0.2 mm. Every process was one-pass processing, and the whole process was carried out under cold water. It was important to note that processing direction (PD), transverse direction (TD) and normal direction (ND) denoted the processing, transverse and normal direction of the sheet, respectively. The K-type thermocouple was embedded in the position of advancing side (AS) and retreating side (RS) to measure the real-time processing temperature and obtain the thermal cycle curve.

### 2.2. The Microstructure and Mechanical Properties Analysis

After the experiment, the sample was cutalong the processing surface. The sample was first ground with sandpaper and then mechanically polished to a mirror. Optical image used Barker reagent HBF_4_ solution (4%) on the surface of electrolytic corrosion 60 s, forming a layer of oxide film, and the polarization. Electron backscattered diffraction (EBSD) analysis was performed using a scanning electron microscope (SEM, Sigma 300, Carl Zeiss AG in Oberkochen, Germany) equipped with EBSD, and the accelerating voltage was 20 KV. In order to obtain EBSD samples, mechanical polishing was carried out first, and then electro polishing in alcohol perchlorate solution (HClO_4_:C_2_H_5_OH= 1:9) at −10 °C for 25 s. Statistics by the grain number of not less than 1000. Step size was 0.3 μm. Equivalent diameter method was used to estimate the average grain size [24], at the same time tested unilaterally up Schmid factor (SF, MAX = 0.5). For TEM testing, the thickness of the sample was ground to 50 μm and further treated by electrolytic double injection technology. The distribution of the second phase particles was observed under transmission electron microscope (TEM, JEOL JEM 2100F).The processing was divided into five parts, of which SZ is divided into three parts, namely the area close to AS (or RS) and the center zone, which were referred to as stirring zone-1 (SZ-1), SZ-2 and SZ-3. The two sides were simply called AS and RS, including TMAZ and HAZ. Moreover, a tensile samplewas taken at each position, as shown in Figure 1b. Figure 1c shows the optical morphology of HRS-SFSP, showing the location of each region. Micro Vickers hardness tester (HVT-1000) was used to measure the hardness value every 0.5 mm from the position 1mm downward from the section to obtain the hardness distribution. The pressure holding time was 10 s and the load was 200 g. The tensile tests were conducted at room temperature using an electronic universal testing machine (Zwick/Z2.5) at a strain rate of 0.01 mm/s. Considering the size of the processing was lesser, the tensile samples were used as shown in Figure 1d [25]. The length of the parallel part between them was 1mm. Such a small size tensile specimen was also applicable to other processing fields [26]. The distribution of the second phase particles was observed by TEM.

## 3. Results

### 3.1. Processing Thermal Cycle Curve

**Figure 2:**
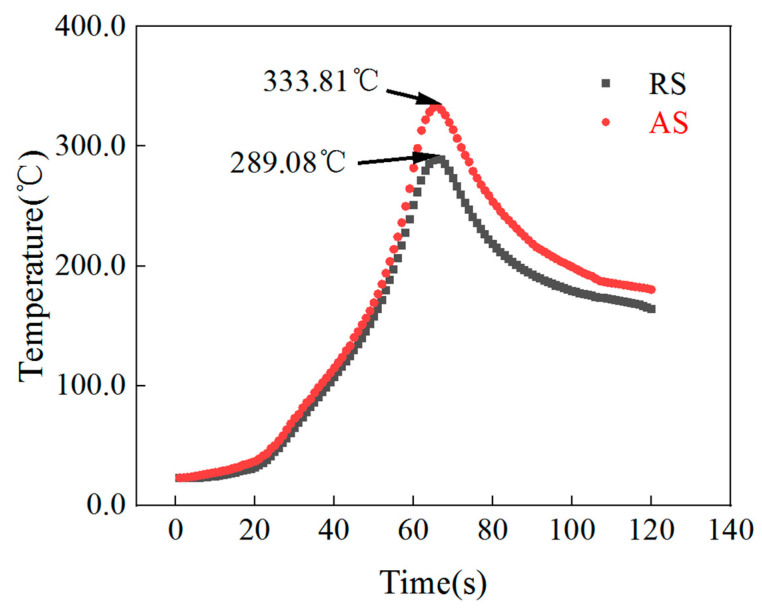
Thermal cycle curves of AS and RS.

Figure 2 shows the thermal cycle curves of AS and RS. It can be seen that the peak temperatures of AS and RS are 68% and 63% of BM, respectively, which are higher than the recrystallization temperature and lower than the T*_m_* of aluminum, and met the best temperature zone for FSP (0.6T*_m_*–0.7T*_m_*), which proved that the processing parameters meet the basic requirements of FSP [27].

### 3.2. Mechanical Property

**Figure 3:**
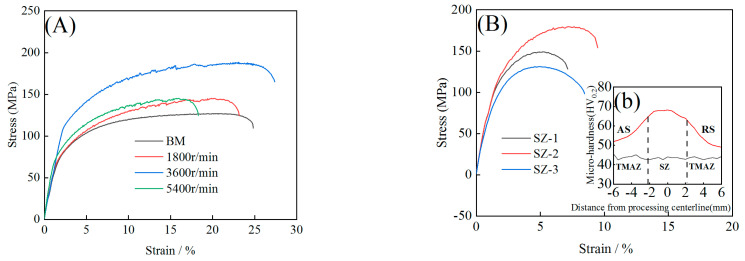

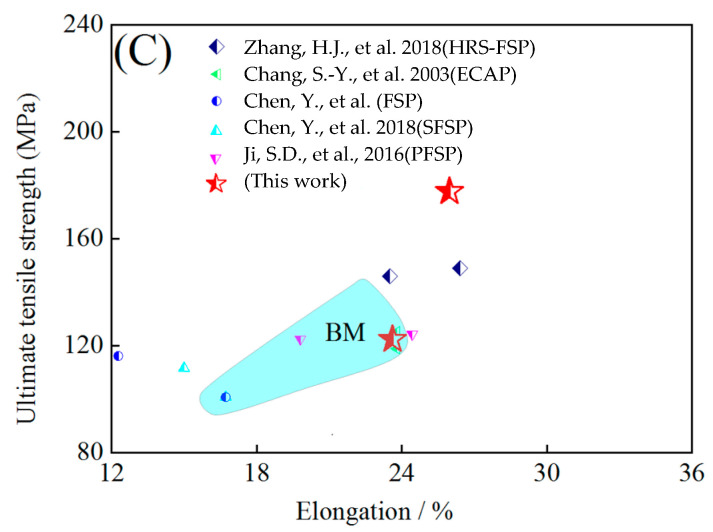
(**A**) Engineering strain curves of different processing parameters; (**B**) Engineering stress-strain curve of different zone in 3600 r/min; (**b**) micro-hardnessand (**C**) references comparison [13,28,29,30].

Figure 3 shows the engineering stress–strain curves and hardness distribution of the aluminum alloy after HRS-SFSP. As can be seen from Figure 3A, the best mechanical properties occur when the rotation speed is 3600 r/min. Moreover, the strength and ductility of the aluminum alloy after HRS-SFSP were increased by 48.7% and 10.2% respectively compared with that of BM. The tensile test was carried out at different positions of the sample with processing parameter of 3600 r/min, and the results are shown in Figure 3B. SZ-2 has the maximum ultimate tensile strength (UTS) of 179.2 ± 3.3 MP and RS has the minimum tensile strength of 119.9 ± 2.9 MPa. It can be seen from Figure 3Bb that the hardness of the processing zone is generally increased. The distribution of the whole hardness presents a “Λ” shape, but the left and right are not completely symmetrical. Figure 3C shows the performance comparison between HRS-SFSP and other references [13,28,29,30]. It was found that HRS-SFSP has the best performance in both strength and ductility. These results show that the difference in mechanical properties of the processing zone formed by HRS-SFSP was caused by the inhomogeneity structure, which improves the strength and ductility of the aluminum alloy.Therefore, it is necessary to further study the microstructure of the processing zone.

### 3.3. EBSD Analysis

#### 3.3.1. Grain Size and Misorientation Angle Distribution Statistics

**Figure 4:**
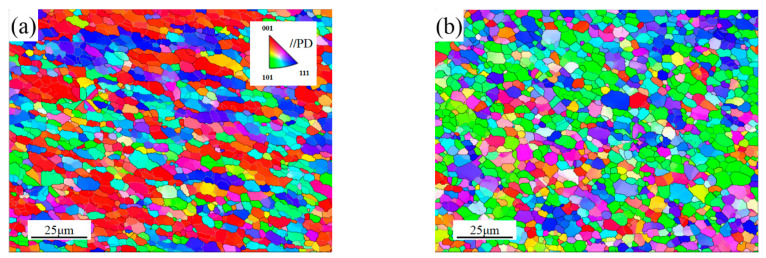

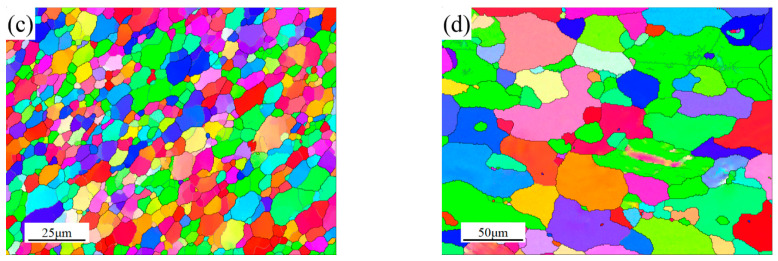
Grain morphology maps of SZ (**a**) SZ-1; (**b**) SZ-2; (**c**) SZ-3 and (**d**) BM.

**Figure 5:**
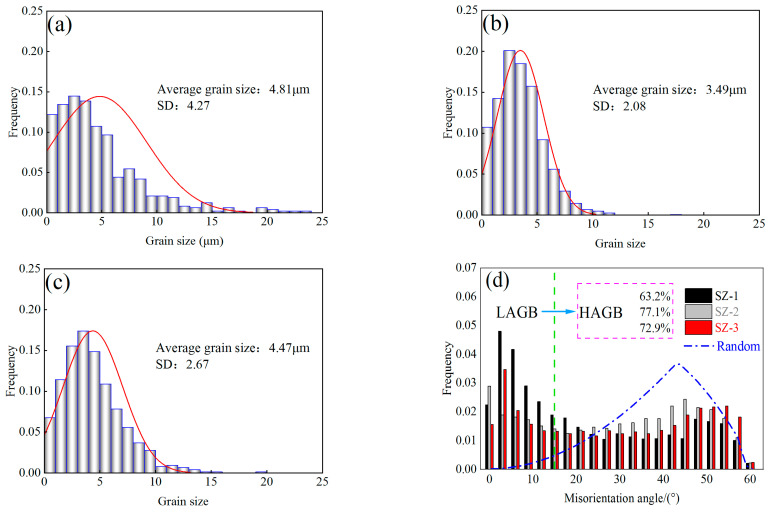
(**a**–**c**) Average grain size and (**d**) misorientation angle distributions of SZ-(1–3).

Figure 4 shows the grain morphology maps of SZ-1, SZ-2 and SZ-3 obtained by EBSD technology: equiaxed grain was observed at all three locations. The black thick line and white thin line in the figure represent HAGBs and LAGBs, respectively. The statistics of grain size and MAD at three locations are shown in Figure 5. It can be seen that there is a difference in grain size from SZ-1 to SZ-3, and this difference is due to the inhomogeneity shear action of the pin tool. It can be seen that there is a difference in grain size from SZ-1 to SZ-3, and SZ-2 has the smallest grain size (3.49 μm). At the same time, the standard deviation (SD) of SZ-2 is the smallest, indicating that the homogeneous degree of SZ-2 grains is the best. *θ*_c_ represents the misorientation angle distribution (MAD). Moreover, *θ*_c_ = 15° was used as the boundary between high angle grain boundaries (HAGBs) and low angle grain boundaries (LAGBs). As can be seen from the MAD of (Figure 5d), the proportion of large angle grain boundaries (HAGBs) in the three regions is also significantly different, of which the largest proportion of SZ-2 is 77.1%, while the smallest proportion of SZ-1 is 63.2%.

#### 3.3.2. Qualitative and Quantitative Statistics of Texture

**Figure 6:**
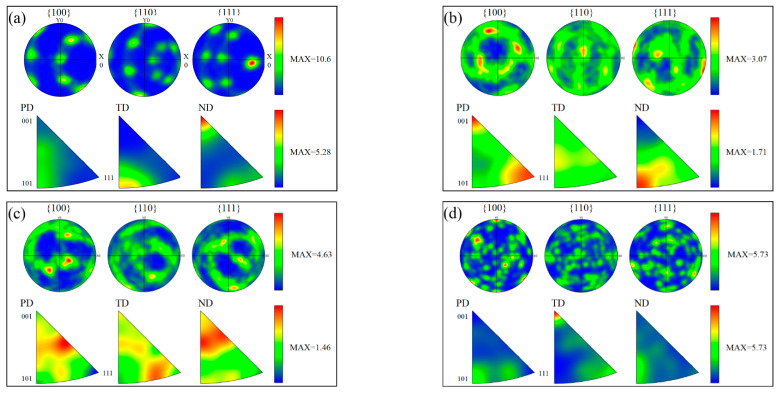
The pole figures and inverse pole figures of (**a**) SZ-1; (**b**) SZ-2; (**c**) SZ-3 and (**d**) BM.

**Figure 7:**
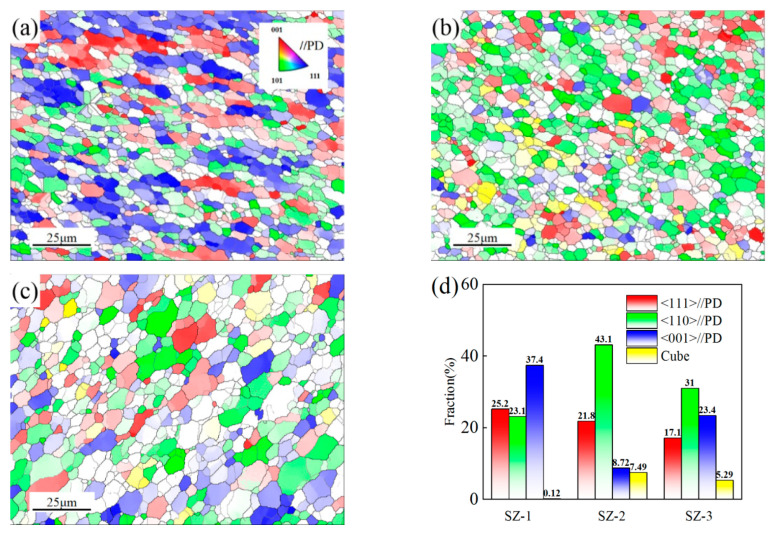
Texture contents of (**a**) SZ-1; (**b**) SZ-2; (**c**) SZ-3 and (**d**) texture statistics under various conditions.

The pole figure (PF) maps in Figure 6 show that the sharper texture of SZ-1, SZ-2 and SZ-3 appear on the different crystal planes. It has the strongest texture in SZ-1 region (MAX = 10.06), while the texture density in SZ-2 region is the weakest (MAX = 3.07). The inverse pole figure (IPF) maps show the same change law as the PF. Figure 7a–c shows the distribution of main textures of SZ-1, SZ-2 and SZ-3. The texture content of the three positions is quite different. Meanwhile, SZ-1 and SZ-3 had the strongest texture density in <001>//PD, while SZ-2 had the strongest texture density in <110>//PD. In addition, Cube texture appears in SZ, which was generally considered the recrystallization texture of high stacking fault energy (SFE) metals (such as aluminum) [7]. In order to identify the texture of each grain, the main texture components were studied, and the criterion of error was set as 10°. Red, green, blue and yellow were used to represent <111>//PD, <110>//PD, <001>//PD and Cube texture, respectively. The results are shown in Figure 7d. The appearance of texture is due to the shear action of pin tool, and the direction of texture was parallel to the shear direction. The difference of texture type and content was due to the inconsistency of fluidity between SZ-1 and SZ-3, which was caused by the different extrusion force of the materials on both sides. Because the extrusion force on the SZ-1 was greater, the texture density was more obvious. Generally, Cube texture was considered the texture formed when aluminum recrystallizes. However, the content of cube texture in SZ was low because of the iron rich particles in 6061 aluminum alloy (Table 1). Iron rich particles can inhibit the formation of cubic texture due to the two theories of “ON” and “OG” [31].

#### 3.3.3. Dislocation Distribution in Grain-Based and Kernel-Based Level

**Figure 8:**
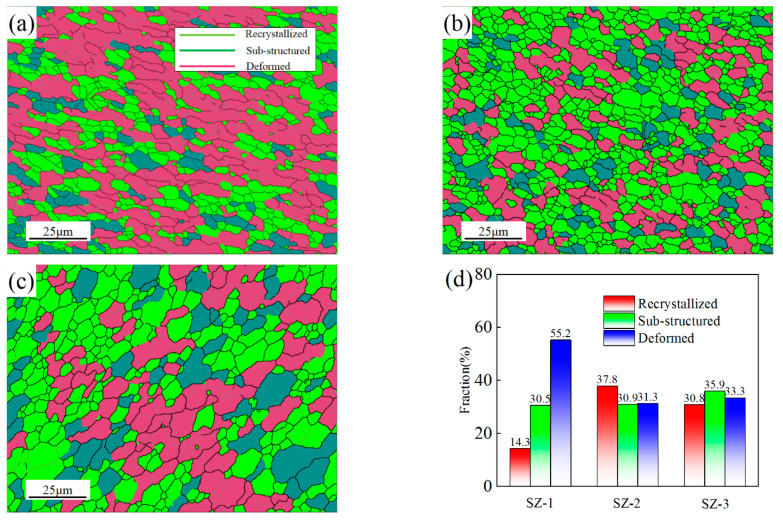
GOS maps of (**a**) SZ-1; (**b**) SZ-2; (**c**) SZ-3 and (**d**) Grain type statistics.

Figure 8 shows the GOS maps of SZ-1, SZ-2 and SZ-3. GOS represents the deviation between the average orientation difference of each grain and the overall average orientation difference. Mitsche et al. [32] has proved that GOS was the best way to distinguish the degree of deformation (or lattice distortion) of grains. According to the value of GOS, the grains can be divided into three categories: recrystallization grains, substructure and deformed structure. For aluminum alloy, it is reasonable to use 0–1.8°, 1.8–3° and 3–15° to represent these three kinds of grains, respectively [33]. According to the statistics, the proportion of recrystallized grains were 14.3%, 37.8% and 35.9%, respectively. These results show that recrystallization occurred in SZ-1, SZ-2 and SZ-3 (the formation mode of recrystallization will be described in detail in Section 3.3.5). The degree of recrystallization growth and deformation is also different under the influence of temperature and pin tool force.

**Figure 9:**
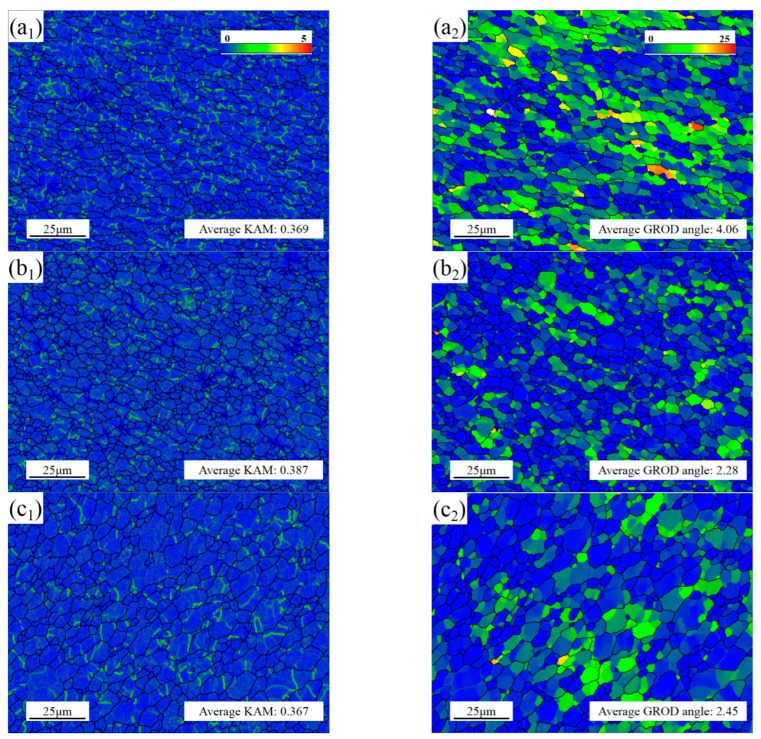
KAM and GROD angle maps of (**a_1_**,**a_2_**) SZ-1; (**b_1_**,**b_2_**) SZ-2 and (**c_1_**,**c_2_**) SZ-3.

**Figure 10:**
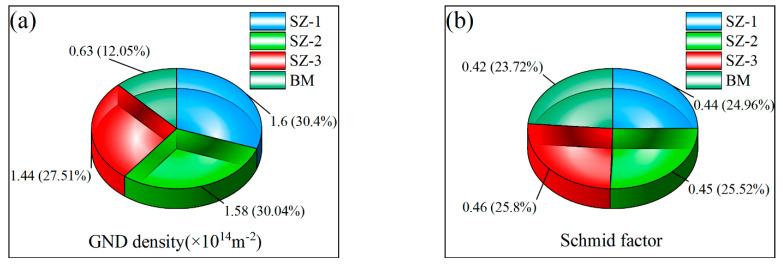
(**a**) GND density and (**b**) SF of SZ-1, SZ-2 and SZ-3.

For plastic deformation, the degrees of deformation were directly related to kernel average misorientation (KAM) and grain reference orientation deviation (GROD) angle, as shown in Figure 9. KAM represented the average misorientation between the center point and other surrounding points, which depended on the size of geometrically necessary dislocation (GND) [34]. The GROD angle describes the orientation deviation of adjacent points in each grain when the average orientation difference is given [35]. The maximum GROD angle value was proportional to dislocation density. Therefore, the phenomenon of sudden rise of GROD angle often occurred in the places where the dislocation accumulates, these dislocation-enriched areas indicated static recrystallization (SRX). Although the recrystallization makes the dislocation density in the grain lower, the dislocation proliferation and movement cause the overall dislocation density to be at a higher level under the vigorous stirring, as shown in Figure 10a. SF was an important index to predict the deformation capacity under external force. As an FCC metal, the slip surface and slip direction of aluminum were (111) and <110>, respectively, and there were 12 slip combinations. The slip system was activated once the SF value was greater than the critical resolved shear stress (CRSS) [36]. After calculation, the SF values of SZ-1, SZ-2 and SZ-3 were 0.44, 0.45 and 0.46 respectively, as shown in Figure 10b. This result shows that all positions in SZ have similar initial deformation capacity, and are higher than BM (0.42).

#### 3.3.4. Processing Boundary Analysis

**Figure 11:**
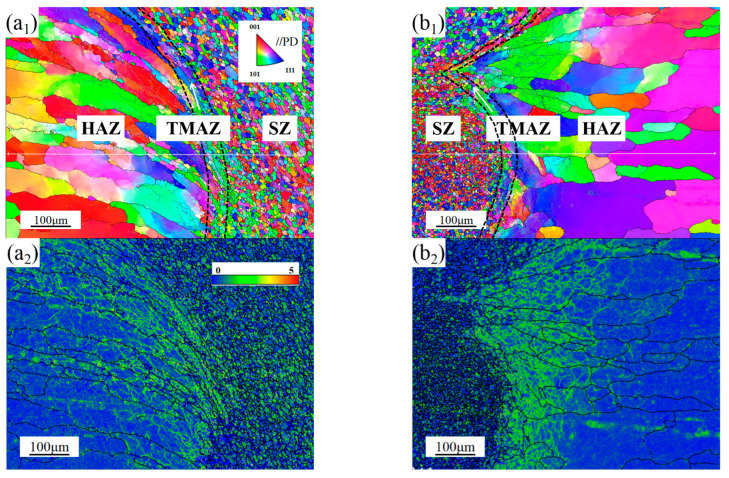

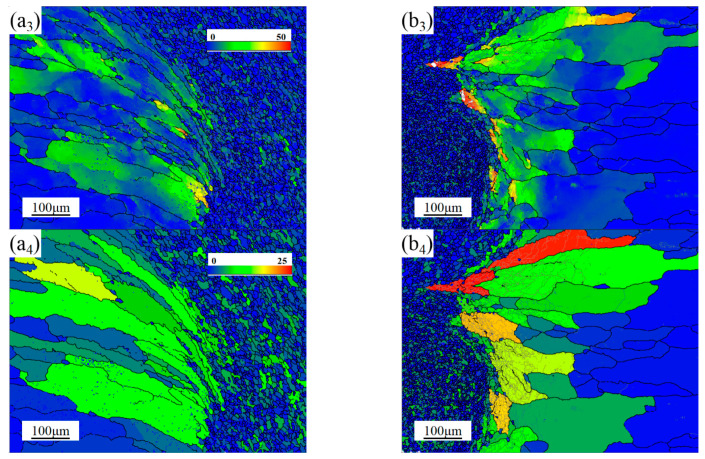
IPF, KAM, GROD angle and GOS maps of (**a_1_**–**a_4_**) AS and (**b_1_**–**b_4_**) RS.

Figure 11 shows the processing boundary in AS and RS. It was not difficult to find that in AS and RS, fibrous grains were significantly refined, grains in TMAZ were partially serrated and some micro regions with low deformation appear in the grains. These results indicated the occurrence of recrystallization process. It could be seen from the GROD angle maps that there were high MAD at the grains of TMAZ, which could become nucleation sites under the condition of solid-phase heating. The distribution of SF shows that the microstructure of AS and RS were inhomogeneous, resulting in a sharp decrease in elongation. HAGBs were mainly distributed in SZ and LAGBs are mainly distributed in TMAZ, which were the results of recrystallization.

#### 3.3.5. Further Analysis of SZ-2 Microstructure

**Figure 12:**
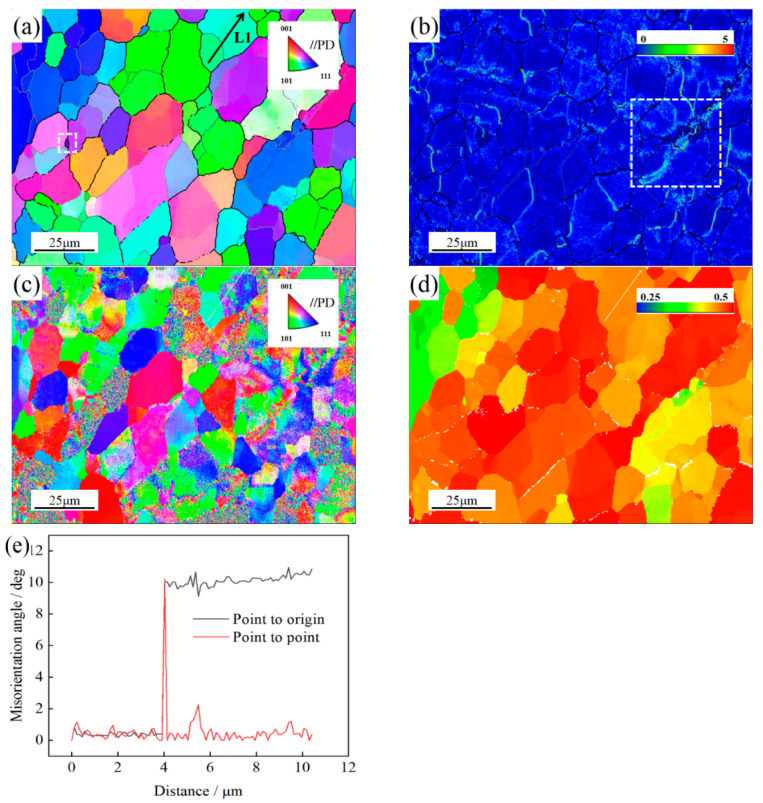
Maps in the SZ-2 of (**a**) IPF; (**b**) KAM; (**c**) GROD axis; (**d**) SF and (**e**) misorientation profiles obtained along the lines marked in Figure 12a: L1.

Compared with SZ-1 and SZ-3, SZ-2 has the best microstructure and properties. Therefore, it is necessary to further analyze the deformation of the microstructure of SZ-2. Figure 12 summarizes the microscopic information of SZ-2 position obtained at a smaller scale (step size = 0.1 μm) using EBSD technology.

The deformed grains in SZ-2 can be analyzed from two aspects: deformation mode and deformation ability. On the one hand, the MAD of grains was the intuitive embodiment of deformation. For SZ-2, the change of MAD was considered the result of recrystallization. Recrystallization can be divided into dynamic recrystallization (DRX) and Static recrystallization (SRX), while DRX can be divided into discontinuous dynamic recrystallization (DDRX) and continuous dynamic recrystallization (CDRX) [37]. Generally, CDRX was the continuation of dynamic response (DRV); it was transformed from LAGBs to HAGBs [38]. Due to the compatibility of deformation between adjacent grains, the grain boundaries are serrated, and fine DRX grains can be observed along these grain boundaries. The serrated grain boundary usually has a high local orientation or strain gradient, so it is a potential location for DDRX nucleation through grain boundary expansion. At the same time, due to the energy difference between adjacent grains in the deformation process, the original grain boundaries migrate locally. The misorientation near the serrated grain boundary gradually changes. Subsequently, due to dislocation rearrangement, LAGBs developed and eventually developed into HAGBs. When HAGBs form a closed loop, fine DRX grains develop and eventually grow into new grains within a sufficient deformation period, as shown in Figure 12a. This is a typical DDRX characterized by grain boundary expansion. It is worth noting that some new fine grains can be observed in the original grains, such as the L1 line segment in Figure 12a. Figure 12e shows the point-to-origin (cumulative) and point-to-point misorientation difference of L1 line segment. It can be seen that the point-to-origin (cumulative) misorientation difference exceeds 10°. Since there are no shear bands or twins at this position, this indicates that the CDRX nucleation mechanism characterized by progressive sub grain rotation occurs in the deformed grains [39]. While there are no sub grains in some grains, these positions prove the existence of SRX. These results indicate that CDRX, DDRX and SRX coexist in SZ-2. In addition, in SZ-2, it was also found that some residual strains were generated due to insufficient DRX, such as the rich region of LAGBs in the white rectangular frame in Figure 12b. This residual stress caused the increase of local dislocation density. However, under the action of external force, the large grains around can quickly consume these dislocations and avoid the generation of cracks.

On the other hand, the deformation capacity reflected the plastic toughness of the material, and GROD axis and SF were the rotation system in the deformation process and important indicators to measure the deformation capacity respectively [40]. Corresponding to GROD angle, GROD axis indicated the axial deviation of each point in a single grain from the average value, while SF described the deformation capacity of each grain. Figure 12c shows the GROD axis map of SZ-2. It can be clearly seen that <100>, <110>, <111> exist in the same grain at the same time, which indicates that the grain deformation of SZ-2 was inhomogeneous in the process of HRS-SFSP.

### 3.4. TEM Analysis

**Figure 13:**
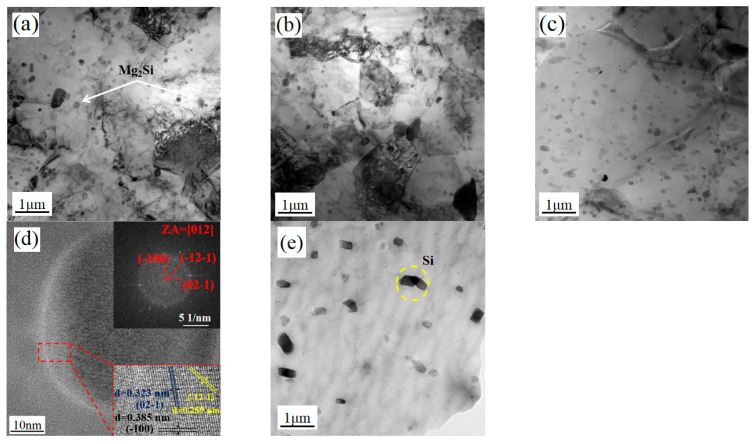
Shows the TEM microstructure of HRS-SFSP, and (**a**–**c**) are the grain and second phase distribution of SZ-1, SZ-2 and SZ-3, respectively; (**d**) HRTEM of Mg_2_Si and (**e**) BM.

**Figure 14:**
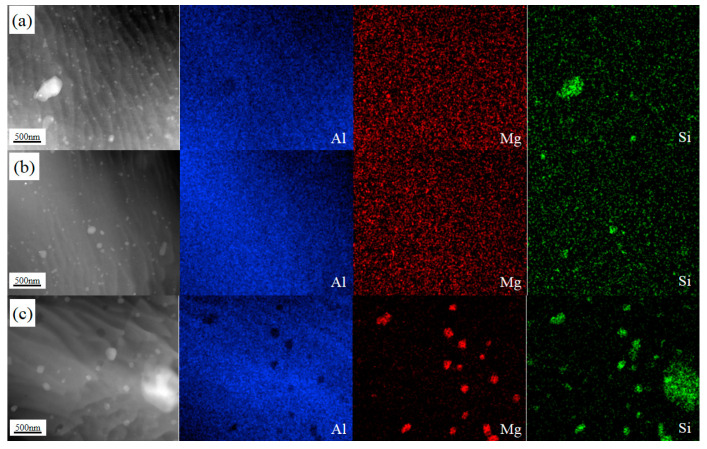
STEM-HAADF and EDS of (**a**) SZ-1; (**b**) SZ-2 and (**c**) SZ-3.

Figure 13 shows the TEM microstructure of HRS-SFSP, and (a)–(c) are the grain and second phase distribution of SZ-1, SZ-2 and SZ-3, respectively. It can be seen that the grains and the second phase at the three positions are significantly refined compared to BM. For 6xxx Al-Mg-Si alloy, the aging precipitation sequence is as follows: supersaturated solid solution → Mg/Si atomic cluster → GP region →β″→β′→β (Mg_2_Si). There are a large number of circular second phase particles in SZ-1 and SZ-2, which were generally considered to be stable β phase [41]. In SZ-3, light colored short rod particles are more common, which are considered not to be completely coarsened β′ phase [42], which was caused by the low temperature on the RS. Figure 13d also shows β high-resolution transmission electron microscope (HR-TEM). Figure 14 shows the distribution of aluminum, Mg and Si of SZ-1, SZ-2 and SZ-3 obtained by high-angle annular dark-field scanning transmission electron microscope (HAADF-STEM). It can be seen that Mg and Si elements in SZ-1 and SZ-2 were evenly distributed, while partial aggregation occurred in SZ-3. These results show that the dispersion of the second phase particles of SZ-3 is the worst. The existence of the second phase will hinder the movement of dislocations. Therefore, the second phase promotes the plastic–elastic interface effect.

## 4. Discussion

### 4.1. Microstructure Evolution in the Processing Zone

The microstructure evolution in the processing zone is the main reason to reveal the improvement of mechanical properties [1,43]. Obviously, recrystallization occurred in SZ. Although the deformed grains in SZ-1 are dominant, the recrystallized grains in SZ-2 and SZ-3 maintain a high proportion (Figure 8). Recrystallized grains provide enough plasticity for aluminum alloy, so it shows good tensile ductility.

For SZ, recrystallization can be divided into dynamic recrystallization (DRX) and Static recrystallization (SRX), while DRX can be divided into discontinuous dynamic recrystallization (DDRX), continuous dynamic recrystallization (CDRX) and geometric dynamic recrystallization (GDRX) [37]. SRX is greatly inhibited due to the rapid cooling of water. GDRX is described as the formation of equiaxed grains by the migration of HAGBs to form serrations and thinning of the grain thickness [44]. With the increase of tool rotation speed, the welding temperature gets higher and the motion of grain boundary gets easier, which can prompt the occurrence of GDRX [45]. Meanwhile, as SZ-1 and SZ-3 are close to the boundaries of processing, they are affected by compression and elongation caused by large strain. Therefore, GDRX can be clearly seen in SZ-1 and SZ-3. Among them, the GDRX of SZ-1 is significantly more than that of SZ-3. This is caused by the difference of material fluidity on both sides. These results indicate that GDRX, CDRX and DDRX coexist in SZ. In addition, the recrystallization mechanism is dominated by GDRX and CDRX in SZ-1 and SZ-3, while CDRX and DDRX are dominant in SZ-2.

### 4.2. The Mechanism of Strengthen

In all kinds of plastic deformation processes, yield strength (YS) has always been an important reference index. The main factors affecting the YS were grain boundary, dislocation, and the size and distribution of the second phase. It is necessary to discuss these influencing factors separately, to determine their effects in the strength of aluminum alloys. Therefore, a comprehensive model was introduced to discuss the contribution of various factors to YS, as shown in Equation (1) [46]:

(1) $$\sigma_{YS}=\sigma_{H-P}+\sigma_{dis}+{M\tau }_{tot}$$

where σ*_H-P_* is boundary strengthening, σ*_dis_* is dislocation strengthening, τ*_tot_* is composed of σ_0_ (CRSS strengthening), σ*_ss_* (solid solution strengthen) and σ*_phase_* (precipitation strengthen). The σ_0_ of aluminum is 9 MPa [38]. These strengthening mechanisms are discussed below.

#### 4.2.1. Grain Boundary Strengthening

Grain boundary strengthening is one of the important strengthening means in plastic deformation. Grain boundary strengthening generally conforms to Hall-Petch relationship, as shown in Equation (2):

(2) $$\sigma_{H-P}=K\cdot d^{-1/2}$$

where K is a constant and d is the average grain size. The K value of aluminum is generally considered to be 70 MPa·μm^1/2^ [47]. The boundary strengthening values of SZ-1, SZ-2 and SZ-3 were 37.4 MPa, 35.2 MPa and 30.5 MPa, respectively.

#### 4.2.2. Dislocation Strengthening

It has been pointed out that the MAD of grains greater than 1.8° is a conventional grain boundary, and less than 1.8° belongs to the category of sub grain, which is the main cause of dislocation. σ*_dis_* is generally described by Equation (3) [46]:

(3) $$\sigma_{dis}=M\alpha Gb\cdot \rho_{dis}^{\frac{1}{2}}$$

where *M* is Taylor factor (TF), α is a constant (0.24) [48], *G* is the shear modulus (26 GPa) [49], b is the Burgers vector (0.286 nm) and *ρ_dis_* is the dislocation density. *ρ_dis_* can be expressed as the GND, which can be described by Equation (4) [50]:

(4) $$\rho^{GND}=\frac{2\Delta \theta_{i}}{ub}$$

where, Δ*θ*_i_ is KAM, u is the step size (0.3 μm), and *b* is the Burgers vector. The GND values are shown in Figure 10.

The slip ability can reflect the ductility. SF and TF were used to describe the slip phenomenon in polycrystals. Generally, SF was mainly used to judge which slip systems start first, while TF represents the ability of slip, and the two were negatively correlated. The SF shown in Figure 10 have made a preliminary judgment on the ductility of each position. However, SF only reflects the plastic deformation of single crystal and cannot determine the interaction between adjacent grains. In solid materials, the interaction between grains may change the original orientation [51]. TF was usually represented by M, which was 3.07 in pure aluminum without texture [52]. However, after HRS-SFSP, texture appears in SZ, so that M without texture cannot be used directly. Therefore, it was necessary to make statistics of its main texture components and content. The texture types and contents of SZ-1, SZ-2 and SZ-3 were given in Table 2, and M is calculated by using the Equation (5) [52]:

(5) $$M=\underset{i}{\sum }f_{i}M_{i}+\left(1-\underset{i}{\sum }f_{i}\right)M_{TF}$$

where *f_i_* is the number fraction of the grain with the same orientation, *M_i_* is the homologous *TF* and *M_TF_* was the *TF* of the rest. Here, we directly used channel 5 analyze software to calculate *TF*. Through calculation, the dislocation strengthening values of SZ-1, SZ-2 and SZ-3 were 6.4 MPa, 6.6 MPa and 6.2 MPa, respectively. It means that dislocation strengthening was not obvious. There were two main reasons: on the one hand, heat input provided the driving force of dislocation movement, which makes the dislocation move continuously and merge and disappear; on the other hand, the shear action of pin tool forms recrystallization, resulting in the disappearance of a large number of dislocation sources and low dislocation density. Therefore, the contribution of dislocation strengthening was very low. In addition, the decrease of dislocation density was also the main reason for the decrease of hardness, as shown in Figure 3B.

#### 4.2.3. Precipitation Phase Strengthening

Generally, the main strengthening method in the alloy is the second phase strengthening. Because 6061 aluminum alloy contains many kinds and quantities of the second phase, and its calculation is very complex, Bardel et al. [53] proposed a model for calculating the second phase, as shown in Equation (6):

(6) $$\sigma_{phase}=2\mathrm{MGb}\beta /L$$

where M is the TF, G is the shear modulus, and b is the Burgers vector, β is a constant (0.28) [53] and L is the average spacing of precipitates. L measured by TEM figures were 168 nm, 159 nm and 176 nm, respectively. The precipitation strengthening of SZ-1, SZ-2 and SZ-3 were 69.7 MPa, 76.8 MPa and 68.7 MPa, respectively.

#### 4.2.4. Solid Solution Strengthening

The content of solid solution elements can be reflected by the content of precipitated phase. The relative volume fraction of precipitates was defined as the ratio of the volume fraction of a sample to the volume fraction of the peak aging state of the BM. In order to simplify the calculation, the volume fraction was replaced by the area fraction. The strengthening effect of solid solution elements on the matrix can be expressed by Equation (7) [54]:

(7) $$\sigma_{ss}=\sum_{j}K_{j}\cdot C_{j}^{2/3}$$

where *C_j_* is the content of solid solution alloy elements and *K_j_* is the corresponding scale factor. For 6061 aluminum alloy, Mg_2_Si has been proved to be the main strengthening phase, in which the *K_j_* values of Mg and Si are 66.3 and 29.0 MPa/wt.%^2/3^ [55], respectively. The calculation results show that the solid solution strengthening of SZ-1, SZ-2 and SZ-3 were 13.7 MPa, 25.5 MPa and 8.44 MPa, respectively. It means that the overall solid solution strengthening of HRS-SFSP is not obvious and more second phase particles were precipitated under the rapid cooling of water.

**Figure 15:**
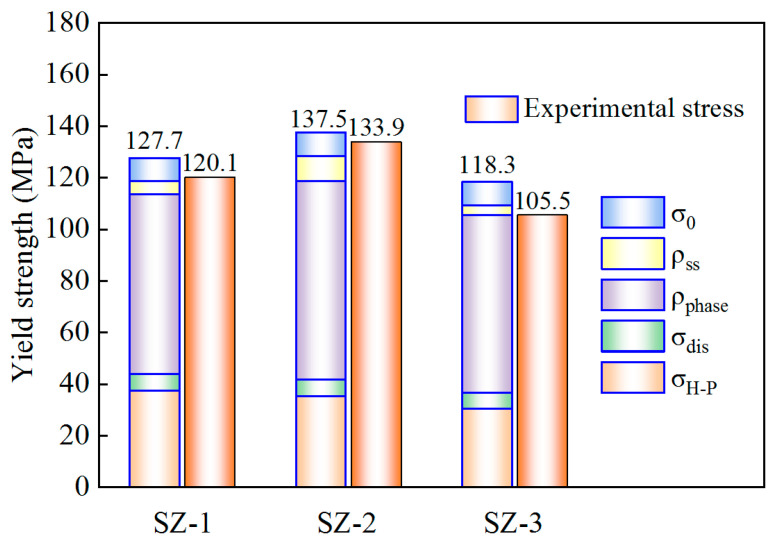
Comparison between experimental stress and calculated stress.

It can be seen from Figure 15 that the calculated YS shows that there were certain differences in the strengthening contributions of SZ-1, SZ-2 and SZ-3, and the YS of SZ-2 was the largest, which was consistent with the conclusion of experimental intensity. Because the strength contribution model was a simple linear superposition, and in fact, there was a role of one side and the other side between these strengthening contributions, the calculated value of YS is always slightly higher than that of the experiment.

### 4.3. The Synergy of Strength and Ductility

**Figure 16:**
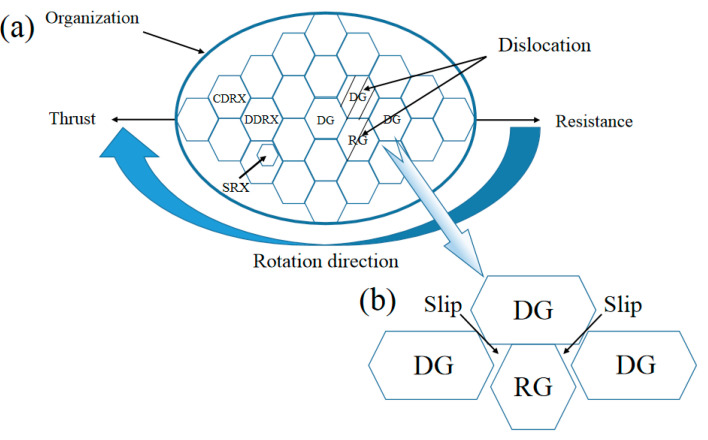
Schematic diagram of (**a**) HRS-SFSP process organization evolution and (**b**) plastic–elastic interfaces.

The schematic diagram of HRS-SFSP process organization evolution is summarized in Figure 16. The recrystallization modes in the SZ were mainly DDRX, CDRX and a small amount of SRX. Due to the low processing temperature, the dynamic recovery and dynamic recrystallization in the thermal deformation stage will consume some dislocation energy, and the thermal energy of SRX was insufficient, resulting in the insufficient driving force of SRX, which was the result of cold water. The rapid cooling of water hinders the subsequent thermal cycling effect. Therefore, the time of static recrystallization was short to avoid excessive grain growth due to subsequent thermal cycle. Because the recrystallization process consumes dislocations, the dislocation density inside was low, while in the deformed grain it was relatively high, which indicated that a recrystallized grain (RG) was sandwiched between two deformed grains (DG), as shown in Figure 16. During uniaxial tension, plastic deformation occurred in recrystallization, while elastic deformation occurred in deformed grains. The difference in deformation caused the synergistic effect of biaxial stress and strain gradient at the plastic–elastic interfaces [56,57]. Biaxial stress activates more slip systems in recrystallization, which strengthened the interaction between dislocations. The strain gradient formed a blank area between recrystallized and deformed grains, as shown in Figure 16b, which led to the accumulation of dislocation density. Therefore, this synergistic effect led to the good ductility of 6061 aluminum alloy after HRS-SFSP under good strength.

## 5. Conclusions

In this paper, HRS-SFSP technology is proposed to balance the strength ductility synergistic effect of aluminum alloys. From a systematic study of the microstructure evolution of the processing zone, the following conclusions could be drawn:

1. HRS-SFSP processing zone shows excellent mechanical properties and overcomes the contradiction between strength and ductility. The strength and ductility of 6061 aluminum alloy are increased by 48.7% and 10.2% respectively compared with BM. This was credited to the plastic–elastic interface generated by the inhomogeneous structure in the processing zone.
2. The plastic–elastic interface is composed of deformed grains and recrystallized grains. Deformed grains produce dislocation gradients by affecting the distribution of dislocations and promote the initiation of more slip systems. Meanwhile, the recrystallization proportions in SZ-1, SZ-2 and SZ-3 are 14.3%, 37.8% and 35.9% respectively, which provide sufficient plasticity for aluminum alloy. These can facilitate strain hardening and improved ductility and are composed of deformed grains and recrystallized grains. Deformed grains produce dislocation gradients by affecting the distribution of dislocations and promote the initiation of more slip systems. Meanwhile, the recrystallization proportions in SZ-1, SZ-2 and SZ-3 are 14.3%, 37.8% and 35.9% respectively, which provide sufficient plasticity for aluminum alloy. These can facilitate strain hardening and improved ductility.
3. The recrystallization mechanism at different locations in the processing zone is different. GDRX and CDRX are dominant in SZ-1 and SZ-3, while CDRX and DDRX are dominant in SZ-2. The transformation of recrystallization mechanism is also an important factor affecting the evolution of microstructures.
4. The difference of mechanical properties in the processing zone comes from the difference of grain size, dislocation density, second phase distribution and solid solubility. The grain boundary strengthening and the second phase strengthening are the main ones. Meanwhile, the uniformly distributed and fine second phase promotes the plastic–elastic interface effect.
5. HRS-SFSP is a modified version of FSP. Although the use of small size tools may affect the processing efficiency, the mechanical properties of aluminum alloys are significantly improved. Therefore, it is recommended to apply it in industry.

## Figures and Tables

**Table 1:** Chemical compositions of 6061 aluminum sheets (massfraction/%).

Element	Mg	Si	Fe	Mn	Cu	Al
content	1.16	0.66	0.4	0.2	0.22	Bal.

**Table 2:** Taylor factor and main texture types and contents contained in SZ-1, SZ-2 and SZ-3.

Location	ψ_1_	Φ	ψ_2_	Number Fraction	Taylor Factor
SZ-1	0°	0°	0°	0.032	2.813
	35°	45°	0°	0.083	
	90°	35°	45°	0.007	
	0°	45°	0°	0.019	
SZ-2	0°	0°	0°	0.045	2.933
	35°	45°	0°	0.030	
	90°	35°	45°	0.048	
	0°	45°	0°	0.044	
SZ-3	0°	0°	0°	0.028	2.907
	35°	45°	0°	0.021	
	90°	35°	45°	0.084	
	0°	45°	0°	0.024	

## Data Availability

Not applicable.

## References

[1] Abbasi M., Givi M., Bagheri B. (2020). New method to enhance the mechanical characteristics of Al-5052 alloy weldment produced by tungsten inert gas. Proc. Inst. Mech. Eng. Part B J. Eng. Manuf..

[2] Mao D., Meng X., Xie Y., Yang Y., Xu Y., Qin Z., Chang Y., Wan L., Huang Y. (2022). Strength-ductility balance strategy in SiC reinforced aluminum matrix composites via deformation-driven metallurgy. J. Alloys Compd..

[3] Moradi M., Nili-Ahmadabadi M., Poorganji B., Heidarian B., Parsa M., Furuhara T. (2010). Recrystallization behavior of ECAPed A356 alloy at semi-solid reheating temperature. Mater. Sci. Eng. A.

[4] Ashouri S., Nili-Ahmadabadi M., Moradi M., Iranpour M. (2008). Semi-solid microstructure evolution during reheating of aluminum A356 alloy deformed severely by ECAP. J. Alloys Compd..

[5] Donatus U., Thompson G., Momoh M., Maledi N., Tsai I.-L., Ferreira R.O., Liu Z. (2018). Variations in stir zone and thermomechanically affected zone of dissimilar friction stir weld of AA5083 and AA6082 alloys. Trans. Nonferrous Met. Soc. China.

[6] Shen Z., Ding Y., Chen J., Amirkhiz B.S., Wen J., Fu L., Gerlich A. (2019). Interfacial bonding mechanism in Al/coated steel dissimilar refill friction stir spot welds. J. Mater. Sci. Technol..

[7] Zhang Y., Jiang J., Wang Y., Xiao G., Liu Y., Huang M. (2021). Recrystallization process of hot-extruded 6A02 aluminum alloy in solid and semi-solid temperature ranges. J. Alloys Compd..

[8] Mishra R., Mahoney M., McFadden S., Mara N., Mukherjee A. (1999). High strain rate superplasticity in a friction stir processed 7075 Al alloy. Scr. Mater..

[9] Peng Y., Zhang Q., Wen L., Xie Z., Huang B., Hu S., Tang H., Wei C. (2022). An Investigation into Microstructures and Mechanical Properties of 1060 Pure Aluminum during Submerged Friction Stir Processing at a High Rotating Speed. Metals.

[10] Li J., Zhao M., Jin L., Wang F., Dong S., Dong J. (2021). Simultaneously improving strength and ductility through laminate structure design in Mg–8.0Gd–3.0Y-0.5Zr alloys. J. Mater. Sci. Technol..

[11] Han M.-S., Lee S.-J., Park J.-C., Ko S.-C., Woo Y.-B., Kim S.-J. (2009). Optimum condition by mechanical characteristic evaluation in friction stir welding for 5083-O Al alloy. Trans. Nonferrous Met. Soc. China.

[12] Sharma D.K., Badheka V.J., Patel V., Upadhyay G. (2021). Recent Developments in Hybrid Surface Metal Matrix Composites Produced by Friction Stir Processing: A Review. J. Tribol..

[13] Zhang H.J., Wang M., Zhu Z., Zhang X., Yu T., Wu Z.Q. (2018). Nugget Structure Evolution with Rotation Speed for High-Rotation-Speed Friction-Stir-Welded 6061 Aluminum Alloy. J. Mater. Eng. Perform..

[14] Abdollahzadeh A., Bagheri B., Abassi M., Kokabi A.H., Moghaddam A.O. (2021). Comparison of the Weldability of AA6061-T6 Joint under Different Friction Stir Welding Conditions. J. Mater. Eng. Perform..

[15] Lin P.-T., Liu H.-C., Hsieh P.-Y., Wei C.-Y., Tsai C.-W., Sato Y.S., Chen S.-C., Yen H.-W., Lu N.-H., Chen C.-H. (2021). Heterogeneous structure-induced strength-ductility synergy by partial recrystallization during friction stir welding of a high-entropy alloy. Mater. Des..

[16] Chen J., Bao C., Ma Y., Chen Z. (2017). Distribution control of AlN particles in Mg-Al/AlN composites. J. Alloys Compd..

[17] Xie Y., Meng X., Mao D., Qin Z., Wan L., Huang Y. (2021). Homogeneously Dispersed Graphene Nanoplatelets as Long-Term Corrosion Inhibitors for Aluminum Matrix Composites. ACS Appl. Mater. Interfaces.

[18] Guru P., Khan F., Panigrahi S., Ram G.J. (2015). Enhancing strength, ductility and machinability of a Al–Si cast alloy by friction stir processing. J. Manuf. Process..

[19] Jiang H., Liu C., Zhang B., Xue P., Ma Z., Luo K., Ma M., Liu R. (2017). Simultaneously improving mechanical properties and damping capacity of Al-Mg-Si alloy through friction stir processing. Mater. Charact..

[20] Lambiase F., Derazkola H.A., Simchi A. (2020). Friction Stir Welding and Friction Spot Stir Welding Processes of Polymers—State of the Art. Materials.

[21] Mohamed I.F., Lee S., Edalati K., Horita Z., Hirosawa S., Matsuda K., Terada D. (2015). Aging Behavior of Al 6061 Alloy Processed by High-Pressure Torsion and Subsequent Aging. Met. Mater. Trans. A.

[22] Khalaf H.I., Al-Sabur R., Abdullah M.E., Kubit A., Derazkola H.A. (2022). Effects of Underwater Friction Stir Welding Heat Generation on Residual Stress of AA6068-T6 Aluminum Alloy. Materials.

[23] Tamjidy M., Baharudin B.T.H.T., Paslar S., Matori K.A., Sulaiman S., Fadaeifard F. (2017). Multi-Objective Optimization of Friction Stir Welding Process Parameters of AA6061-T6 and AA7075-T6 Using a Biogeography Based Optimization Algorithm. Materials.

[24] Seo P., Kang C. (2005). The effect of raw material fabrication process on microstructural characteristics in reheating process for semi-solid forming. J. Mater. Process. Technol..

[25] Liu X.-C., Zhen Y.-Q., Sun Y.-F., Shen Z.-K., Chen H.-Y., Guo W., Li W.-Y. (2020). Local inhomogeneity of mechanical properties in stir zone of friction stir welded AA1050 aluminum alloy. Trans. Nonferrous Met. Soc. China.

[26] Çam G., Yeni Ç., Erim S., Ventzke V., Koçak M. (1998). Investigation into properties of laser welded similar and dissimilar steel joints. Sci. Technol. Weld. Join..

[27] Yi D., Mironov S., Sato Y.S., Kokawa H. (2016). Effect of cooling rate on microstructure of friction-stir welded AA1100 aluminum alloy. Philos. Mag..

[28] Chen Y., Jiang Y., Zhang F., Ding H., Zhao J., Ren Z. (2018). Water Cooling Effects on the Microstructural Evolution and Mechanical Properties of Friction-Stir-Processed Al-6061 Alloy. Trans. Indian Inst. Met..

[29] Chang S.-Y., Lee K.-S., Choi S.-H., Shin D.H. (2003). Effect of ECAP on microstructure and mechanical properties of a commercial 6061 Al alloy produced by powder metallurgy. J. Alloys Compd..

[30] Ji S.D., Meng X.C., Ma L., Gao S.S. (2016). Effect of groove distribution in shoulder on formation, macrostructures, and mechanical properties of pinless friction stir welding of 6061-O aluminum alloy. Int. J. Adv. Manuf. Technol..

[31] Doherty R., Kashyap K., Panchanadeeswaran S. (1993). Direct observation of the development of recrystallization texture in commercial purity aluminum. Acta Met. Mater..

[32] Mitsche S., Poelt P., Sommitsch C. (2007). Recrystallization behaviour of the nickel-based alloy 80 A during hot forming. J. Microsc..

[33] Jiang J.-F., Wang Y., Liu Y.-Z., Xiao G.-F., Li H. (2021). Microstructure and mechanical properties of 7005 aluminum alloy processed by one-pass equal channel reciprocating extrusion. Trans. Nonferrous Met. Soc. China.

[34] Gussev M., Leonard K. (2019). In situ SEM-EBSD analysis of plastic deformation mechanisms in neutron-irradiated austenitic steel. J. Nucl. Mater..

[35] Shen R.R., Ström V., Efsing P. (2016). Spatial correlation between local misorientations and nanoindentation hardness in nickel-base alloy 690. Mater. Sci. Eng. A.

[36] Jiang S., Jia Y., Wang X. (2020). In-situ analysis of slip transfer and heterogeneous deformation in tension of Mg-5.4Gd-1.8Y-1.5Zn alloy. J. Magnes. Alloys.

[37] Sakai T., Miura H., Goloborodko A., Sitdikov O. (2009). Continuous dynamic recrystallization during the transient severe deformation of aluminum alloy 7475. Acta Mater..

[38] Heidarzadeh A., Chabok A., Pei Y. (2019). Friction stir welding of Monel alloy at different heat input conditions: Microstructural mechanisms and tensile behavior. Mater. Lett..

[39] Xie B., Zhang B., Ning Y., Fu M. (2019). Mechanisms of DRX nucleation with grain boundary bulging and subgrain rotation during the hot working of nickel-based superalloys with columnar grains. J. Alloys Compd..

[40] Zhang Y., Jiang S., Wang S., Sun D., Hu L. (2017). Influence of partial static recrystallization on microstructures and mechanical properties of NiTiFe shape memory alloy subjected to severe plastic deformation. Mater. Res. Bull..

[41] Sauvage X., Bobruk E., Murashkin M., Nasedkina Y., Enikeev N., Valiev R. (2015). Optimization of electrical conductivity and strength combination by structure design at the nanoscale in Al–Mg–Si alloys. Acta Mater..

[42] Sato Y.S., Kokawa H., Enomoto M., Jogan S. (1999). Microstructural evolution of 6063 aluminum during friction-stir welding. Met. Mater. Trans. A.

[43] Bagheri B., Alizadeh M., Mirsalehi S.E., Shamsipur A., Abdollahzadeh A. (2022). Nanoparticles Addition in AA2024 Aluminum/Pure Copper Plate: FSSW Approach, Microstructure Evolution, Texture Study, and Mechanical Properties. Jom.

[44] Huang K., Logé R. (2016). A review of dynamic recrystallization phenomena in metallic materials. Mater. Des..

[45] Yu P., Wu C., Shi L. (2021). Analysis and characterization of dynamic recrystallization and grain structure evolution in friction stir welding of aluminum plates. Acta Mater..

[46] Kamikawa N., Huang X., Tsuji N., Hansen N. (2009). Strengthening mechanisms in nanostructured high-purity aluminium deformed to high strain and annealed. Acta Mater..

[47] Yu C., Kao P., Chang C. (2005). Transition of tensile deformation behaviors in ultrafine-grained aluminum. Acta Mater..

[48] Hansen N., Huang X. (1998). Microstructure and flow stress of polycrystals and single crystals. Acta Mater..

[49] Xie M., Huan Q., Li F. (2020). Quick and repeatable shear modulus measurement based on torsional resonance using a piezoelectric torsional transducer. Ultrasonics.

[50] Yan Z., Wang D., He X., Wang W., Zhang H., Dong P., Li C., Li Y., Zhou J., Liu Z. (2018). Deformation behaviors and cyclic strength assessment of AZ31B magnesium alloy based on steady ratcheting effect. Mater. Sci. Eng. A.

[51] Mi Y., Nie H., Wang T., Li X., Hao X., Liang W. (2019). Effect of Anisotropy on Microstructures and Mechanical Properties of Rolled Ti/Al/Mg/Al/Ti Laminates. J. Mater. Eng. Perform..

[52] Starink M., Wang S. (2003). A model for the yield strength of overaged Al–Zn–Mg–Cu alloys. Acta Mater..

[53] Bardel D., Perez M., Nelias D., Deschamps A., Hutchinson C., Maisonnette D., Chaise T., Garnier J., Bourlier F. (2013). Coupled precipitation and yield strength modelling for non-isothermal treatments of a 6061 aluminium alloy. Acta Mater..

[54] Myhr O.R., Grong Ø., Andersen S.J. (2001). Modelling of the age hardening behaviour of Al–Mg–Si alloys. Acta Mater..

[55] Zeng X., Xue P., Wu L., Ni D., Xiao B., Ma Z. (2019). Achieving an ultra-high strength in a low alloyed Al alloy via a special structural design. Mater. Sci. Eng. A.

[56] Wu X., Jiang P., Chen L., Yuan F., Zhu Y.T. (2014). Extraordinary strain hardening by gradient structure. Proc. Natl. Acad. Sci. USA.

[57] Wu X.L., Jiang P., Chen L., Zhang J.F., Yuan F.P., Zhu Y.T. (2014). Synergetic Strengthening by Gradient Structure. Mater. Res. Lett..

